# Quality Traits of “Cannabidiol Oils”: Cannabinoids Content, Terpene Fingerprint and Oxidation Stability of European Commercially Available Preparations

**DOI:** 10.3390/molecules23051230

**Published:** 2018-05-20

**Authors:** Radmila Pavlovic, Giorgio Nenna, Lorenzo Calvi, Sara Panseri, Gigliola Borgonovo, Luca Giupponi, Giuseppe Cannazza, Annamaria Giorgi

**Affiliations:** 1 CRC-Ge.S.Di.Mont.—Centre for Applied Studies in the Sustainable Management and Protection of the Mountain Environment, CRC-Ge.S.Di.Mont.—Università degli Studi di Milano, Via Morino 8, Edolo, 25048 Brescia, Italy; radmila.pavlovic1@unimi.it (R.P.); Luca.giupponi@unimi.it (L.P.); anna.giorgi@unimi.it (A.G.); 2 Department of Agricultural and Environmental Sciences—Production, Landscape, Agroenergy, Università degli Studi di Milano, Via Celoria 2, 20133 Milan, Italy; lorenzocalvi@yahoo.it; 3 UPFARM-UTIFAR—The Professional Union of Pharmacists for Orphan Medicines; Piazza Duca d'Aosta, 14, 20124 Milan, Italy; giorgio@farmacianenna.it; 4 Department of Health, Animal Science and Food Safety, Università degli Studi di Milano, Via Celoria 10, 20133 Milan, Italy; 5 Dipartimento di Scienze per gli Alimenti, la Nutrizione e l’Ambiente, Università degli Studi di Milano, Via Celoria 2, 20133 Milan, Italy; gigliola.borgonovo@unimi.it; 6 Dipartimento di Scienze della Vita, Università di Modena e Reggio Emilia, Via Campi 103, 41121 Modena, Italy; giuseppe.cannazza@unimore.it

**Keywords:** cannabidiol, CBD oil, terpenes, hemp seed oil, GC-MS, HPLC-Q-Exactive-Orbitrap-MS

## Abstract

Cannabidiol (CBD)-based oil preparations are becoming extremely popular, as CBD has been shown to have beneficial effects on human health. CBD-based oil preparations are not unambiguously regulated under the European legislation, as CBD is not considered as a controlled substance. This means that companies can produce and distribute CBD products derived from non-psychoactive hemp varieties, providing an easy access to this extremely advantageous cannabinoid. This leaves consumers with no legal quality guarantees. The objective of this project was to assess the quality of 14 CBD oils commercially available in European countries. An in-depth chemical profiling of cannabinoids, terpenes and oxidation products was conducted by means of GC-MS and HPLC-Q-Exactive-Orbitrap-MS in order to improve knowledge regarding the characteristics of CBD oils. Nine out of the 14 samples studied had concentrations that differed notably from the declared amount, while the remaining five preserved CBD within optimal limits. Our results highlighted a wide variability in cannabinoids profile that justifies the need for strict and standardized regulations. In addition, the terpenes fingerprint may serve as an indicator of the quality of hemp varieties, while the lipid oxidation products profile could contribute in evaluation of the stability of the oil used as milieu for CBD rich extracts.

## 1. Introduction

Cannabidiol (CBD) and tetrahydrocannabinol (THC) are the most common cannabinoids in medical cannabis preparations [1]. The are both responsible for a variety of pharmacological actions that can have remarkable applications, but unlike THC, CBD does not possess any psychoactive effects [1]. Several studies suggest that CBD can be effective in treating epilepsy and other neuropsychiatric disorders, including anxiety and schizophrenia [2,3,4]. CBD may also be effective in treating post-traumatic stress disorder and may have anxiolytic, antipsychotic, antiemetic and anti-inflammatory properties [5,6,7]. This plethora of pharmacological activities has led to rapid changes in the cultural, social and political legal viewpoints regarding the utilization of cannabis-based preparations [8]. Although there is still a complicated legal milieu that calls for caution, it is undeniable that there is an enormous interest from consumers/patients in the utilization of CBD dietary supplements. This has created an exploding industry of CBD products in Europe and around the world. “CBD enriched oils”, obtained from extraction of different *Cannabis sativa* L. chemotypes with high content of CBD, are the most popular products used [9,10,11,12]. 

Since CBD, in contrast to THC, is not a controlled substance in the European Union [13] several companies produce and distribute CBD-based products obtained from inflorescences of industrial hemp varieties. However, due to the lack of specific regulations, no analytical controls are mandatory for CBD-based products, leaving consumers with no legal protection or guarantees about the composition and quality of the product they are acquiring. Currently, CBD-based products are not subject to any obligatory testing or basic regulatory framework to determine the indication area, daily dosage, route of administration, maximum recommended daily dose, packaging, shelf life and stability. Exceptions are galenical “CBD oil” prepared by pharmacists following medical prescriptions in several European Union countries such as Germany, Italy and Holland. The German Drug Codex (DAC), which is published by the Federal Union of German Associations of Pharmacists (ABDA) and functions as a supplementary book to the Pharmacopoeia, suggests a preparation of 5% CBD in medium chain triglycerides oil also indicating detailed analytical controls of galenic preparations [14].

In Italy medical cannabis represents a multifaceted reality [9,10,11,12]. At present Dutch Bedrocan varieties (Bedrocan, Bediol, Bedica and Bedrolite as representative) [15] and the new strain FM2 produced by Military Pharmaceutical Chemical Works of Florence, Italy (authorized in November 2015 by a Ministerial Decree) can be prescribed to treat a wide range of pathological conditions [16]. Indeed. Italian galenic pharmacies are authorized to prepare precise cannabis doses for vaping, herbal teas, resins, micronized capsules and oils. The oil preparation has received considerable attention since it is easy to adjust the individual administration dose required throughout the treatment period, and due to the enhanced bioavailability of its active compounds [9,10,11,12]. Among abovementioned strains, Bedrolite with CBD and THC contents of 9% and <1%, respectively, is frequently used for the preparation of galenic “CBD-based oil”. Anyway, pharmacies are also allowed to distribute CBD oils obtained from hemp, but declared as additives or aromatic preparations, if produced in Italy or designed as dietary supplement if imported from other European countries.

*Cannabis sativa* L. has been cultivated throughout the world for industrial and medical purposes. The European Union permits the cultivation of plants for hemp products based on the THC content being less than 0.2%. EU Regulation 1307/2013 [17] states that hemp farmers are required to use seeds of cannabis varieties included in the European Union catalogue. In general, specialized extraction procedures, among which the most common is supercritical CO_2_ extraction, are used to draw out an extract rich in CBD from the cannabis to obtain CBD oil formulations [18,19]. This product also contains other biological active compounds such as omega-3 fatty acids, vitamins, terpenes, flavonoids and other phytocannabinoids like cannabichromene (CBC), cannabigerol (CBG), cannabinol (CBN) and cannabidivarian (CBCV) [10,11,12]. 

Among non-cannabinoids compounds, special attention must be paid to terpenes that represent the largest group (more than 100 different molecules) of cannabis phytochemicals [20,21,22,23]. Monoterpenes, diterpenes, triterpenes and sesquiterpenes are important components present in the cannabis resin responsible for its unique aromatic properties. Due to their ability to easily cross cell membranes and the blood-brain barrier, they can also influence the medicinal quality of different cannabis chemotypes [24]. Several therapeutic approaches based on the combined use of cannabinoids and terpenes have been developed recently. Particularly, treatment of sleeping disorders and social anxiety by adding caryophyllene, linalool and myrcene to CBD/THC extracts gave encouraging results [25]. In addition, differences between the pharmaceutical properties of diverse C*annabis sativa* L. varieties have been attributed to strict interactions, defined as ‘entourage effects’, between cannabinoids and terpenes as a result of synergic action [25]. Recently Pagano et al. [26] investigated the different effect of a pure CBD preparation versus a standardized *Cannabis sativa* extract with the same concentration of cannabidiol (CBD) in the remission of mucosal inflammation in a mouse models of colitis. The author reveled that under the same experimental conditions, pure CBD just partially ameliorated colitis, while *Cannabis sativa* L. extract almost entirely reduced the injuries. These findings sustain the rationale of the ‘entourage effect’ achievable by combining CBD with other minor *Cannabis* constituents. 

The quality of *Cannabis* macerated oils has already been investigated in previous research demonstrating the importance of selecting correct preparation methods and conditions as well as studying the evolution of major and minor compounds (cannabinoid and terpenes) during storage in order to define the ideal shelf-life and management guidelines (storage temperature) [12]. Oxidation products derived from fatty acid degradation during the storage period of macerated oils are critical for overall formulation stability [27,28]. Galenic preparations are usually prepared by using pharmacopeia grade olive oil (FU) to minimise the formation of large quantities of aldehydes and ketones that can also influence the digestibility of the macerated oil [9,12,29].

Since the production of CBD-based oils as dietary supplements has increased rapidly, and since they are frequently used for therapeutic purposes, the main scope of this study was to assess the overall quality of 14 CBD oil preparations produced in different European countries and purchased on the Internet and highlight possible criticisms. Moreover, a Bedrolite macerated oil prepared as a galenic product was used as a reference therapeutic formulation. In order to define and increase knowledge about the characteristic of CBD oils, an in-depth chemical profiling of cannabinoids, terpenes and oxidation compounds by means of GC-MS and HPLC-Q-Exactive-Orbitrap-MS analytical platforms was presented herein.

## 2. Results and Discussion

### 2.1. Cannabinoids Content

Current ambiguous all-purpose regulations allow huge variations in the quality and safety of the CBD-based preparations available on the market and clear labelling regarding the exact concentration of CBD is not yet mandatory. Our results demonstrate that CBD concentrations were not always in accordance with producer information (Table 1). As a matter of fact, nine out of 14 tested samples presented concentrations that differed notably from the declared amount, while the remaining five preserved CBD levels within optimal limits (the variation was less than 10%). Our analysis also revealed that two preparations (particularly oils 8 and 10) exhibited higher levels of CBD than those specified by producers, while in another two (samples Oil_3 and Oil_14) the CBD content was far inferior to the stated values. In one sample, the theoretical CBD concentration was not indicated on the label and therefore values obtained could not be compared to the producer’s statement. Taken together, the results highlighted the extreme variability of the commercialised CBD oil preparations, justifying the need for stricter regulations/controls. Precise information regarding the composition of each lot that is available on the market is crucial for consumers who have to be able to properly adapt the recommended dose to the available/purchased preparation [9]. These results are in agreement with those obtained from a preliminary study toward the labeling accuracy of cannabidiol extracts preparations from products available on the US market. In the tested products, 26% contained less CBD than labeled, which could negate any potential clinical response [30]. The over labeling of CBD products in the study was similar in magnitude to levels that triggered warning letters to 14 businesses in 2015–2016 from the US Food and Drug Administration suggesting that there is a continued need for federal and state regulatory agencies to take steps to ensure label accuracy of these consumer products.

Although CBD is a principal constituent of the examined cannabis oil extracts, the original plant is only capable of producing its acid form, cannabidiolic acid (CBDA). Decarboxylation of CBDA catalysed by thermal exposure during extraction conditions leads to the conversion of CBDA to the CBD as the corresponding decarboxylated (neutral) counterpart. Therefore, the determination of CBDA is important in order to evaluate the CBDA decarboxylation rate and effectiveness of the reaction during the extraction process [31]. Interestingly, looking through the web sites of the CBD oil producers enrolled in this survey, it can be found that some of them published an analytical report in which only the total CBD content as the sum of CBD + (0.877 CBDA) is reported. This is quite problematic as the biological effects of the neutral and acidic forms are remarkably different [5]. Generally, expressing the CBD content as a sum of the acidic and neutral forms is conditioned by the analytical method applied. Concretely, it occurs when gas chromatography (GC), one of the most commonly used analytical platforms for cannabinoid analysis, is used [31]. It involves the heating of the sample at high temperature in the injector prior to the chromatographic separation that leads inevitably to the decarboxylation of the cannabinoid acids. Therefore, the analytical result is the sum of the acid and neutral forms. The GC method is still officially employed by the authorities for the determination of cannabinoids, but obviously is unsuitable. A few research groups continue to suggest that an accurate cannabinoid profile should be evaluated by determining the acid and neutral forms separately [12,31]. Results obtain in this study confirms this necessity. Employing the LC-HRMS technology, we were able to distinguish the acidic form from neutral CBD, and to examine the wide concentration range. As can be seen in Table 1, in the majority of the samples the CBDA concentration was found to be negligible compared to the amount of CBD (for example, samples Oil_4 and Oil_15). On the contrary, there were a few samples with a significant amount of CBDA. A striking example is Oil_3, in which the CBDA content exceeded CBD, and only the sum of both forms justified the CBD percentage declared by the producer. Furthermore, it is evident that the label concentration of CBD in Oil_12 is reached only when the sum of both forms is considered, bearing in mind the significant amount of CBDA.

Nevertheless, all producers underline that their manufacturing methods yield the so called full spectrum extract, which means that hemp extracts contain different phyto-cannabinoids, including THC, CBN, CBG, THCA, CBGA and others, depending from cannabis strain and extraction method. In order to achieve full-spectrum in a hemp extract, the profile of bioactive compounds that a plant flower contains must be transferred into the extract itself without compromising any aspect of the profile.

In comparison with previous works available on cannabis oil [9,10,11,31] we employed a HRMS method that provided more complete information regarding the cannabinoids profile and amount in the oil composition. Actually, besides CBD as a principal cannabinoid, we were able to detect and to quantify the six most significant cannabinoids, including the essential ones (THC, THCA and CBDA) along with quantification of CBN, CBG and CBGA. The obtained results clearly show that 12 out of 14 samples contained THC which is attention-grabbing because of its potential intoxicating activity. The THC content showed the considerable variability in the analysed samples, but was mainly at the levels describable as low (0.2%) [17]. Only one among all THC-positive sample (Oil_6) contained a considerable amount of THC (0.35%), which is matter of concern because the manufacturer declared the product to be THC-free. This result highlights the importance of also specifying the amount of THC or any another intoxicating cannabinoid present in commercialised CBD oils.

**Figure 1:**
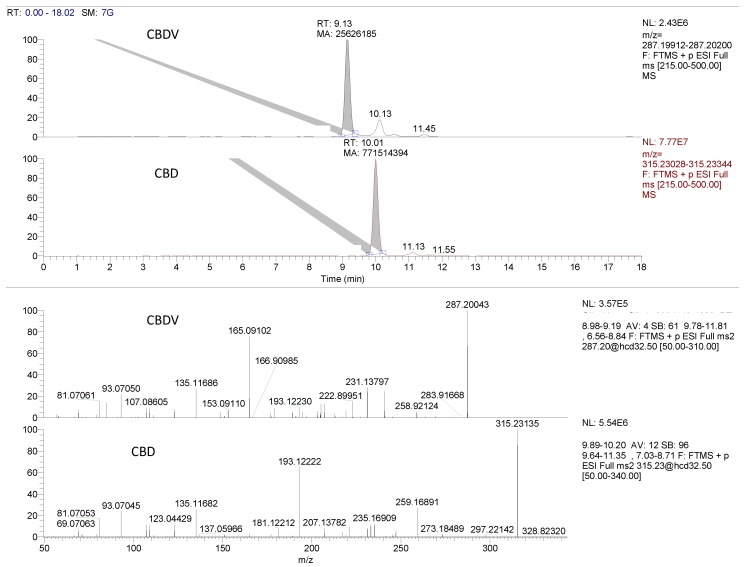
Retrospective data analysis reveals the occurrence of CBDV: full MS-dd-MS^2^ chromatogram and relative fragmentation pattern of parent ion (287.20048) obtained in dd-MS^2^ acquisition mode. For the comparison, the CBD signal and fragmentation pattern is also presented.

CBN was quantifiable in the vast majority of samples (except Oil_14). Its detection is of great importance as it is not considered to be a natural cannabinoid but rather an artefact formed by THC oxidation during plant aging, by use of an inadequate extraction procedure or inappropriate storage conditions [32]. Therefore, its determination may assist in the evaluation of the quality of CBD oils with regards to the raw plant material used, extraction method applied and storage. For example, in the sample Oil_13 the quantity of the CBN was more than twice the amount of THC. Considering CBN as a degradation product of THC, it would be better to think through the sum of THC+CBN as a relevant parameter for the evaluation of initial THC concentration in the oil extract. In addition, CBN, though much less psychoactive than THC, express sedative effects [33,34] which is why its content should be indicated on the label along with THC. It is well known that THC derives from the decarboxylation of tetrahydrocannabinolic acid (THCA) [20], and this is the reason why the amount of THCA was quantified in this study. Our results did not reveal any significant presence of either THCA (Table 1) or cannabigerolic acid (CBGA) which is the precursor of the all other cannabinoid acids. CBGA gives by decarboxylation cannabigerol (CBG) that either was completely absent or present in minor quantities. The quantification of CBGA and CBG did not turn out to be imperative, but their presence could serve as a confirmation that the oil sample contains a natural, full spectrum cannabis extract. Furthermore, employing the retrospective analysis, several other minor “untargeted” compounds were detected by means of the Orbitrap (Thermo Fisher Scientific, San Jose, CA, USA) ^®^ analyser. Among others (data not shown) it is important to highlight the persistent occurrence of CBDV in all analysed samples. Figure 1 shows the fragmentation pattern of CBDV and CBD. Bearing in mind that this compound has expressed significant physiological activity [33] and that accompanies the CBD as its analogue, it should be included in any quality evaluation of full spectrum CBD oil preparations. Besides, we noticed that when the hemp seed oil was used as matrix, the signal of CBDV augments notably, which means that maybe one portion of CBDV derives from hemp oil, not from flower extract [34]. 

Bedrolite oil extract (Oil_1) obtained by a recently published procedure [12], is a defined galenic formulation that has been used for distinct therapeutic purposes. It was included in this study as a “reference material” from a well-defined starting material (cannabis plant variety) and made using a standardized/authorized preparation procedure. There are at least two reasons to use the cannabinoid profile of Bedrolite oil extract as a reference point in the evaluation of CBD-rich hemp oils. Firstly, it can be considered as a *full-spectrum extract* that preserves the natural ratios of cannabinoids, any impurities that can compromise the experiments should be absent. Secondly, many consumers tend to replace galenic oil preparations (such Bedrolite oil extract) with CBD-rich hemp oil extract, due to the fact that a medical prescription is required for the former. Our study revealed that Bedrolite oil extract contains 0.8% of CBD. This is in agreement with theoretical percentage (0.9%) that should be found in the Bedrolite oil extract: the inflorescence contains 9% of CBD and the dilution ratio during the extraction is 1:10. However, as regards the cannabinoids profile (Table 1), it is evident that the quantities of CBDA, THC, THCA and CBGA are inferior compared with CBD-rich hemp oil extracts. These data are of great importance as they highlight the reduced concentration of all cannabinoids in Bedrolite oil extract compared to CBD hemp oil extract.

The reasons for all the abovementioned variations between examined samples are numerous and multiple. The final composition of CBD-rich hemp oil extracts depends on the chemotype and quality of the industrial hemp used, but it is also conditioned by the extraction method applied. Unfortunately, not all producers indicate the extraction method used. Only four declared the use of supercritical CO_2_ fluid extraction, which is shown to be the method of choice in that the low temperature and inert atmosphere results in higher CBD yields [18,19]. However, the main drawback of this technology is its high cost, and it is reasonable to assume that solvent extraction is also used for the inexpensive industrial processing. However, it is questionable if this is a correct choice for a product for human consumption because residual solvents (typically hexane, ethanol, isopropyl alcohol, toluene, benzene, xylene and acetone) may contaminate the final product [32]. Without having complete information on the methods of CBD oils preparation, we investigated the occurrence of the most frequently used extraction solvents as solvent residues. Our analysis revealed the sporadic incidence of acetone (Table 2, ketones section) that is more probably present as a lipid oxidation product rather than as a true residual solvent. Nevertheless, the presence of some volatile compounds that might be considered as problematic impurities from solvents residues was detected (Table 2, miscellaneous section). Namely, the samples Oil_4 and Oil_6 showed the presence of 1,3-dimethylbenzene while in the sample Oil_3, 1,2,4-trimethylbenzene was detected. Those aromatic compounds were not present in galenic preparation (Oil_1).

### 2.2. Volatile Fingerprint: Terpene Profile and Secondary Lipid’s Oxidation Products

**Figure 2:**
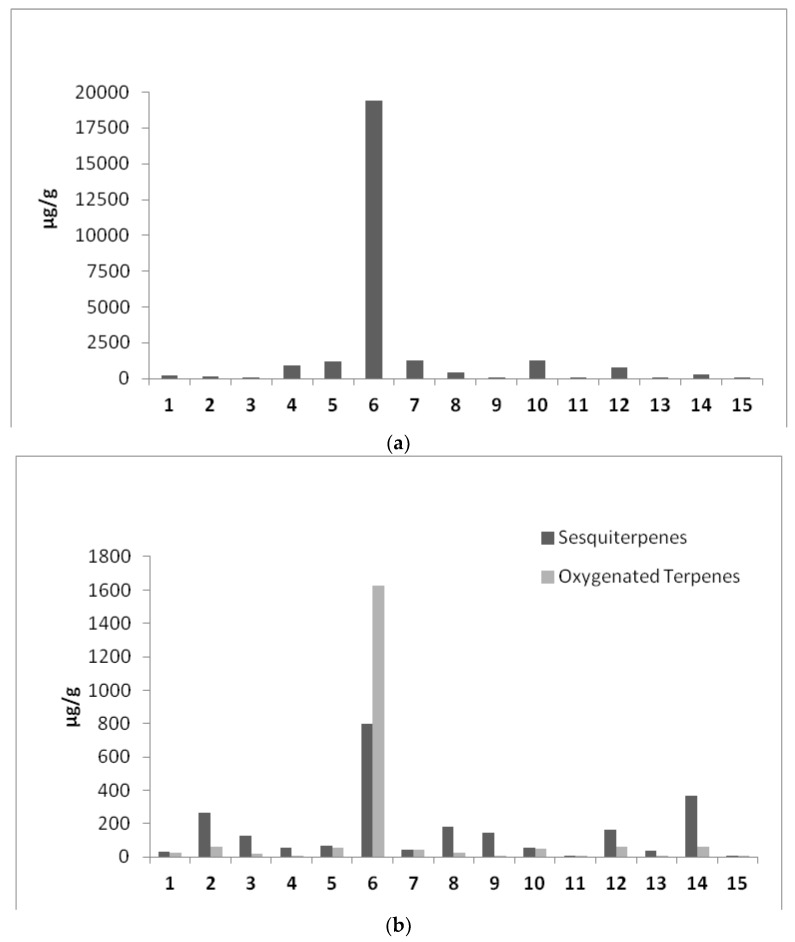
Terpenes classes quantified in CBD based oils preparations (expressed as μg/g IS equivalents) (**a**) Mono/di/tri Terpenes (**b**) sesquiterpenes and oxygenated terpenes.

Terpenes and cannabinoids share biosynthetic pathways and, in fact, cannabinoids are terpenophenolic compounds. In *Cannabis* plants, terpenes are secreted and stored together with cannabinoids in glandular trichomes. Considering this fact also in relation to recent evidence of the synergic action of terpenes and cannabinoids (“entourage effect”), a comprehensive survey of terpenes is fundamental for the evaluation of cannabis oil preparations as dietary supplement with therapeutic applications. Complete data concerning the terpenes profile are summarized and reported in Table 2. Overall, up to 110 volatile compounds composed the volatile fingerprint, including 48 terpenes that are further divided into classes as presented in Figure 2. The sample Oil_6 contained an extremely high amount of terpenes compared with all other samples. α-Pinene, β-myrcene and limonene are the most concentrated terpenes in this preparation, which points toward the extremely efficient extraction method applied. Samples 5, 12 and 14 contained a distinct number of various terpenes, although in far lower concentration compared to sample Oil_6. Apparently, these formulations were obtained by an extraction process able to preserve naturally occurring terpenes profile from initial C*annabis sativa* plants, as their terpene profile is in accordance with those already published in literature [12,21,22,23,24,35]. Similarly, Bedrolite oil extract (Oil_1) contains various terpene structures, reflecting the initial plant profiling. A particular profile is observed for the sample Oil_4 that showed a different terpene fingerprint compared to the other oils as it predominantly contains monoterpene subclass molecules. It has previously been demonstrated how the preparation method used for the production of cannabis extracts is able to affect the presence of different terpenes [18,19], and this is most probably reason of such a specific terpene profile found in Oil_4.

β-Myrcene and limonene accompanied by β-ocimene and *trans*-caryophyllene were found in all samples, but in a much reduced amounts compared to sample 6, and their concentration differed greatly from sample to sample. α-Pinene and β-pinene were identified in the majority of samples, excluding samples Oil_3 and Oil_13. This remains unclear considering that the two pinenes are quite balanced within the different *Cannabis* varieties representing around the 10% of the terpenes group and not exceeding 15–20% [20]. The occurrence of α-terpinolene in all samples (except Oil_2 and Oil_13), might be important as this compound was suggested as a genetic marker for distinguishing two important gene pools for breeding low-THC varieties [35,36]. Sample Oil_14 was particularly rich in *trans*-caryophyllene followed by α-humulene. The dominance of those two sesquiterpenes over the other terpenes detected in this preparation may indicate the geographic provenience of the starting *Cannabis sativa* material, and as a matter of fact, the producer specified the mountain region where the plant was cultivated. 

It is also important to notice that sample Oil_15 was almost completely deprived of a terpene fraction aside from the traces of the main three mentioned above. This might indicate an inefficient/inadequate processing of the starting materials or even artificial addition of CBD to the oil matrix, as some essential cannabinoids were also missing (Table 1). In addition, extremely low terpene content was established for samples 11 and 13, while the remaining samples had total terpene contents in the range between 174 and 1367 μg/g, with pronounced variation in composition from sample to sample. Nevertheless, qualitative and quantitative differences observed in the chemical profiles of terpene fractions are conditioned by many factors such as: hemp variety, cultivation and environmental conditions harvest time and post-harvest conditions, storage and drying of raw plants, extraction procedure applied, matrix used and finally storage of the oil formulation.

Besides hydrocarbon terpenes, oxygenated terpenoids such as linalool and α-terpineol, were found in some preparations (Table 2, Figure 2) with a notably high concentration again in sample Oil_6. Those compounds correspond to secondary photooxidation products of the initial terpenes. In the presence of light and singlet oxygen, terpenes are also known to undergo photooxidation leading to the formation of allylic hydroperoxides [35]. 

In addition to the terpene compound profiles that accounted for more than 90% of the detected volatile constituents of the oils, it was possible to note the presence of other organic compounds commonly found in natural extracts such as esters, alcohols, aldehydes and ketones (Table 2). Only a few low chain esters could actually be identified, with ethyl acetate dominating in sample Oil_3. Its presence is most likely to be due to the preparation method and could also be considered indicative of potential adulteration. 

**Figure 3:**
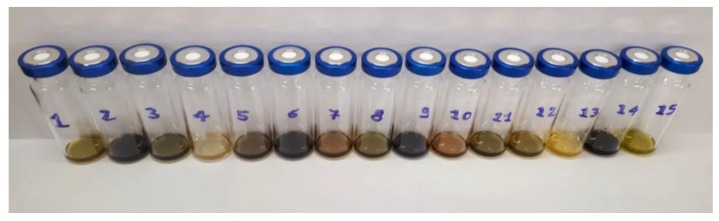
Different colors observed in CBD-based oil products.

On the another hand, the detection of aldehydes and ketones suggests the initiation of lipid peroxidation of polyunsaturated fatty acids (PUFA) in the oils used as a matrix, as demonstrated in our previous work concerning the observed trends of these compounds during storage of macerated *Cannabis*-derived oils [12]. It is well documented that peroxidation of PUFA leads to the formation of a well-defined series of aldehydes and ketones such as nonenal, hexanal and pentanal, 2-heptenal, especially during storage. The rate of formation of lipid oxidation products depends strictly on several factors, among which the most important are the preparation method temperature, fatty acid composition of the oil in which *Cannabis* extract was dissolved and the storage conditions (storage temperature) as recently demonstrated in a study [12]. These parameters are crucial to define the ultimate characteristics of the final products as evidenced also by the color of the samples (Figure 3). Other volatile decomposition compounds frequently encountered include 2-hexenal, 2-octenal, 2,4-nonadienal, 4,5-dihydroxydecenal [37], some of which also appeared to be present in some of our samples. 

**Figure 4:**
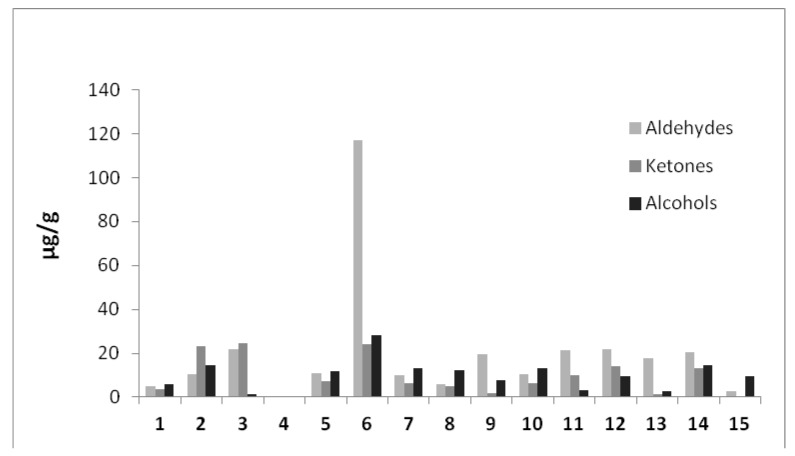
Lipid oxidation products quantified in CBD based oils preparations (expressed as μg/g SI equivalents).

For the CBD oils analyzed in this study, tree different oil typologies were used: medium chain triglycerides (MCT oil, one sample), olive oil (six samples) and hemp seed oil (eight samples). It is worth emphasizing that sample Oil_4 was almost completely deprived of lipid oxidation products (Table 2, Figure 4). This preparation was the only one prepared in MCT oil, which means that this kind of matrix is less susceptible to oxidative degradation than the olive or hemp seed oils declared as matrices for other preparations enrolled herein 

As far as olive oil is concerned, is often used by producers as it has a strong nutritional potential, being rich in the polyunsaturated fatty acids. Moreover, FU oil (pharmaceutical grade olive oil) is used for the preparation of CBD galenic formulations [9,12,29] as it was performed for Bedrolite oil extract. 

Regarding the hemp seed oil used as a carrier for dissolving the CBD extract (hence the term “CBD hemp oil”) some clarifications regarding this kind of preparation are indispensable because misconceptions that may confuse the final users of this preparations still exist. “CBD oil” expression is typically limited to extracts in oil of flowering buds and not stalks, fibers, or seeds of each *Cannabis sativa* L. variety. Hemp seeds do not contain any cannabinoids, but their contact with the resin secreted by the epidermal glands located on flowers, and leaves and/or a bad selection of the bracts of the perigonium can cause the appearance of some cannabinoids in hemp oil [34,38]. Therefore, any cannabinoids detected actually represent hemp seed oil contaminants. Their concentration is influenced by the hemp variety and by the seed cleaning process. Although the cannabinoid concentration in hemp seed oils is usually extremely low, it must be determined before oil commercialization [39]. Nevertheless, the seeds of industrial hemp plants have important uses in human nutrition [40,41] and this is reason why its oil is used as an adequate, naturally resembling matrix for CBD-enriched products. Hemp seed oils represent good sources of protein, and are rich in omega-3 and omega-6 fatty acids with an ideal n3/n6 PUFA nutrition ratio according to WHO guidelines [42,43]. It should be considered that hemp oil is rich in unsaturated fatty acids which are the components susceptible to oxidation phenomena during storage. Although hemp seed oil was shown to be more predisposed to peroxidation than olive oil [44], this study did not identify any significant differences between these two matrixes as far as on-going peroxidation was concerned. Nevertheless, the critical point is to assess stability during the storage period, that is not reported or available for any of the CBD preparations analysed here. This represents a fundamental issue since the formation of lipid oxidation products is related with the decreasing concentration of cannabinoids and terpenes, as well [12]. Therefore, the investigation of trends of compounds characterizing the formulations is essential to define the management conditions. Moreover, an adequate storage temperature would be useful to define the correct expiry date of the products as they are commercialized in EU as dietary supplements.

## 3. Materials and Methods 

### 3.1. Materials

Fourteen samples of commercially available CBDs oil were purchased on the Internet between December 2017 and January 2018. The purchase was based on the main product brands available on the European market. Table 3 summarizes the main characteristics of the samples. Samples were kept at room temperature (as indicated by the manufacturers) before analyses and sample codes (Oils 2–15) were assigned to them in accordance with the order of acquisition. Bedrolite^®^ oil olive extract (assigned as Oil_1) was obtained from a Bedrolite Bedrocan International, Postbus, CA, Veendam, Netherlands^®^ medical *Cannabis* chemotype. Exhaustive analytical procedures were described in details in our recently published article [12].

### 3.2. Chemical and Reagents 

All HPLC or analytical grade chemicals were from Sigma (Sigma–Aldrich, St. Louis, MO, USA). Formic acid 98–100% was from Fluka (Sigma–Aldrich, St. Louis, MO, USA). Ultrapure water was obtained through a Milli-Q system (Millipore, Merck KGaA, Darmstadt, Germany). For head-space (HS) analysis, the SPME coating fiber (DVB/CAR/PDMS, 50/30 μm) was from Supelco (Bellefonte, PA, USA). Acetonitrile, 2-propanol, formic acid LC-MS grade were purchased from Carlo Erba (Milan, Italy). CBD, THC, CBN, CBG, CBNA, THCA, CBGA were purchased from Sigma Aldrich (Round Rock, TX, USA).

### 3.3. Terpenes GC-MS Analysis 

One hundred mg of each oil sample were weighed and put into 20 mL glass vials along with 100 μL of the inyernal standard (IS, 4-nonylphenol, 2000 μg/mL in 2-propanol). Each vial was fitted with a cap equipped with a silicon/PTFE septum (Supelco). A temperature of 37 °C was selected as both as the extraction and equilibration temperature according to previous published research, in order to prevent possible matrix alterations ensuring the most efficient adsorption of volatile compounds onto the SPME fibre [15,16]. To keep the temperature constant during analysis, the vials were maintained in a cooling block (CTC Analytics, Zwingen, Switzerland). At the end of the sample equilibration time (30 min), a conditioned (60 min at 280 °C) SPME fiber was exposed to the headspace of the sample for 120 min using a CombiPAL system injector autosampler (CTC Analytics). All analytical parameters were already validated in our previous research [12].

Analyses were performed with a Trace GC Ultra coupled to a Trace DSQII quadrupole mass spectrometer (MS) (Thermo-Fisher Scientific, Waltham, MA, USA) equipped with an Rtx-Wax column (30 m × 0.25 mm i.d. × 0.25 μm film thickness) (Restek, Bellefonte, PA, USA). The oven temperature program was: from 35 °C, held for 8 min, to 60 °C at 4 °C/min, then from 60 to 160 °C at 6 °C/min and finally from 160 to 200 at 20 °C/min. Helium was the carrier gas, at a flow rate of 1 mL/min. Carry over and peaks originating from the fibres were regularly assessed by running blank samples. After each analysis fibres were immediately thermally desorbed in the GC injector for 5 min at 250 °C to prevent contamination. The MS was operated in electron impact (EI) ionisation mode at 70 eV. An alkanes mixture (C8-C22, Sigma R 8769) was run under the same chromatographic conditions as the samples to calculate the Kovats Retention Indices (RI) of the detected compounds. The mass spectra were obtained by using a mass selective detector, a multiplier voltage of 1456 V, and by collecting the data at rate of 1 scan/s over the *m/z* range of 35–350. Compounds were identified by comparing the retention times of the chromatographic peaks with those of authentic compounds analyzed under the same conditions when available, by comparing the Kovats retention indices with the literature data and through the National Institute of Standards and Technology (NIST) MS spectral database. The quantitative evaluation was performed using the internal standard procedure and the results were finally expressed as μg/g or mg/g IS equivalents of each volatile compounds. All analyses were done in triplicate.

### 3.4. Cannabinoids LC-Q-Exactive-Orbitrap-MS Analysis

The cannabinoids profile and content were evaluated by the procedure recently published by us [12]. Briefly, the oil samples for HPLC-Q-Exactive-Orbitrap-MS analysis were prepared by dissolving 100 mg of each oil in 10 mL of isopropanol. After adding the 1 μg/mL of IS, 10 μL of each sample were diluted in 890 μL of initial mobile phase from which 2 μL was injected. Chromatography was accomplished on an HPLC system (Thermo Fisher Scientific, San Jose, CA, USA) that was made up of a Surveyor MS quaternary pump with a degasser, a Surveyor AS autosampler with a column oven and a Rheodyne valve with a 20 μL loop. Analytical separation was carried out using a reverse-phase HPLC column 150 × 2 mm i.d., 4 μm, Synergi Hydro RP, with a 4 × 3 mm i.d. C18 guard column (Phenomenex, Torrance, CA, USA). The mobile phase used in the chromatographic separation consisted of a binary mixture of solvents A (0.1% aqueous formic acid) and B (acetonitrile). The gradient was initiated with 60% eluent A with a linear decrease up to 95% in 10 min. This condition was maintained for 4 min. The mobile phase was returned to initial conditions at 14 min, followed by a 6-min re-equilibration period (total run time: 20 min). The flow rate was 0.3 mL/min. The column and sample temperatures were 30 °C and 5 °C, respectively. The mass spectrometer Thermo Q-Exactive Plus (Thermo Scientific) was equipped with a heated electrospray ionisation (HESI) source. Capillary temperature and vaporiser temperature were set at 330 and 280 °C, respectively, while the electrospray voltage was adjusted at 3.50 kV (operating in both positive and negative mode). Sheath and auxiliary gas were 35 and 15 arbitrary units, with S lens RF level of 60. The mass spectrometer was controlled by the Xcalibur 3.0 software (Thermo Fisher Scientific). The exact mass of the compounds was calculated using Qualbrowser in Xcalibur 3.0 software. The FS-dd-MS^2^ (full scan data-dependent acquisition) in both positive and negative mode was used for both screening and quantification purposes. Resolving power of FS adjusted on 140,000 FWHM at *m*/*z* 200, with scan range of *m/z* 215–500. The automatic gain control (AGC) was set at 3 × 10^6^, with an injection time of 200 ms. A targeted MS/MS (dd-MS^2^) analysis operated in both positive and negative mode at 35,000 FWHM (*m/z* 200). The AGC target was set to 2 × 10^5^, with the maximum injection time of 100 ms. Fragmentation of precursors was optimised as two-stepped normalised collision energy (NCE) (25 and 40 eV). Detection was based on calculated exact mass of the protonated/deprotonated molecular ions, at least one corresponding fragment and on retention time of target compounds [12]. Extracted ion chromatograms (EICs) were obtained with an accuracy of 2 ppm *m/z* from total ion chromatogram (TIC) engaging the *m/z* corresponding to the molecular ions [M + H]^+^ 315,23145 for CBD and THC, 311,20020 for CBN, 317.24716 for CBG and 311.2024 for CBN. In ESI- the molecular ions [M−H]^−^ considered were 357.2164 for CBDA and THCA, while CBGA was detected by 359,22269. 

## 4. Conclusions

Taken together, the results presented in this study indicate the pronounced variability of CBD concentrations in commercialized CBD oil preparations. The differences found in the overall cannabinoids profiles accompanied with discrepancies revealed for the terpenes fingerprint justify the necessity to provide firmer regulation and control. Precise information regarding CBD oil composition is crucial for consumers, as individual doses throughout the administration period have to be adapted according to CBD bioavailability. This is of fundamental importance regarding consumer safety, as CBD oil preparations are also used in therapeutical purposes, regardless of the fact that they are registered as dietary supplements.

## Figures and Tables

**Table 1:** Cannabinoid content (expressed as % *w*/*w* and in μg/g) in investigated CBD oils (average ± S.D., *n* = 2).

(% *w*/*w*)					Cannabinoids Content (μg/g)													
Samples Code	Deviation from Declared CBD Percentage	Declared CBD ^1^	Revealed CBD ^2^		CBD		THC		CBN		CBG		CBDA		THCA		CBGA	
					Average	±SD	Average	±SD	Average	±SD	Average	±SD	Average	±SD	Average	±SD	Average	±SD
Oil_1 ^3^	9.00	0.9	0.89		8143	170.2	232	4.9	14	0.3	<0.01	/	884	18.8	123	2.6	7	0.1
Oil_2	8.49	4	3.66		36,567	257.3	1908	13.5	208	1.5	716	5.1	42	0.3	1	0.0	12	0.1
Oil_3	21.21	1	0.79		3247	241.7	148	10.5	40	2.8	16	1.1	5282	373.5	191	13.5	693	49.0
Oil_4	15.29	5	4.24		42,352	2395.8	0.01	0.0	3	0.2	<0.01	/	6	0.3	196	11.1	19	1.1
Oil_5	10.53	4	4.42		43,509	3076.6	533	37.7	69	4.9	<0.01	/	802	56.7	17	1.2	27	1.9
Oil_6	4.44	3	2.87		28,536	1008.9	3546	125.4	481	17.0	<0.01	/	152	5.4	29	1.0	8	0.3
Oil_7	8.27	4	4.33		42,601	1807.4	526	22.3	65	2.8	<0.01	/	804	34.1	12	0.5	26	1.1
Oil_8	35.41	3	4.06		39,962	3108.3	695	54.1	62	4.8	<0.01	/	753	58.6	47	3.7	22	1.7
Oil_9	7.63	3	3.23		32,212	683.3	1607	34.1	345	7.3	23	0.5	88	1.9	25	0.5	6	0.1
Oil_10	23.89	4	4.96		48,879	1036.9	557	11.8	79	1.7	<0.01	/	774	16.4	58	1.2	23	0.5
Oil_11	/	/	0.24		1875	68.9	36	1.3	7	0.3	<0.01	/	634	23.3	32	1.2	18	0.7
Oil_12	19.28	2	1.61		12,758	180.4	494	7.0	188	2.7	6	0.1	3862	54.6	107	1.5	97	1.4
Oil_13	36.20	4	2.55		24,444	2419.8	568	56.2	1105	109.4	624	61.8	1229	121.7	27	2.7	22	2.2
Oil_14	38.14	5	3.09		23,186	655.8	524	14.8	67	1.9	460	13.0	8828	249.7	358	10.1	216	6.1
Oil_15	24.33	3	2.27		22,692	320.9	<0.01	/	<0.01	/	5687	80.4	9	0.1	<0.01	/	4	0.1

^1^ CBD declared on labels, ^2^ CBDtot (sum of CBD +0.877 × CBDA); ^3^ Bedrolite oil extract prepared as galenic product—detailed description of the method and its suitability was given previously by Calvi et al., 2018 [12].

**Table 2:** Volatile compounds profile extracted by using HS-SPME and GC/MS from CBD oils samples.

		**Oil Samples**	**1**		**2**		**3**		**4**		**5**		**6**		**7**		**8**	
		**Matrix**	**FU Oil**		**Hemp Seed Oil**		**Olive Oil**		**MCT Oil**		**Olive Oil**		**Hemp Seed Oil**		**Olive Oil**		**Hemp Seed Oil**	
**RI ^a^**	**R.T ^b^**	**Compound**	**Average** **^c^** **μg/g**	**SD** **(±)**	**Average ^c^** **μg/g**	**SD** **(±)**	**Average ^c^** **μg/g**	**SD** **(±)**	**Average ^c^** **μg/g**	**SD** **(±)**	**Average ^c^** **μg/g**	**SD** **(±)**	**Average ^c^** **μg/g**	**SD** **(±)**	**Average ^c^** **μg/g**	**SD** **(±)**	**Average ^c^** **μg/g**	**SD** **(±)**
		***Alcohols***																
831	20.63	1-Hexanol	2.08	0.14	5.15	0.71	n.d.	-	n.d.	-	2.55	0.16	8.10	0.12	2.58	0.03	11.52	0.37
868	21.43	3-Hexen-1-ol	0.66	0.07	0.67	0.11	n.d.	-	0.55	0.02	1.47	0.08	1.46	0.04	1.76	0.05	n.d.	-
849	22.02	2-Hexen-1-ol	n.d.	-	n.d.	-	n.d.	-	n.d.	-	0.77	0.11	n.d.	-	1.10	-	n.d.	-
969	23.07	1-Octen-3-ol	n.d.	-	3.90	0.47	n.d.	-	n.d.	-	0.73	0.15	1.73	0.03	1.09	0.51	n.d.	-
960	23.17	1-Heptanol	n.d.	-	0.83	0.13	n.d.	-	n.d.	-	0.50	0.01	2.49	0.10	n.d.	-	n.d.	-
1059	25.48	1-Octanol	n.d.	-	n.d.	-	1.29	0.11	n.d.	-	0.59	0.03	5.23	0.63	n.d.	-	n.d.	-
1068	27.98	3,3,6-Trimethyl-1,5-heptadien-4-ol	2.39	0.46	n.d.	-	n.d.	-	n.d.	-	n.d.	-	n.d.	-	n.d.	-	n.d.	-
1036	31.59	α-Toluenol	0.42	0.03	2.74	0.59	n.d.	-	n.d.	-	2.36	0.16	3.96	0.47	3.22	0.23	n.d.	-
1136	32.06	Benzeneethanol	0.51	0.04	1.39	0.32	n.d.	-	n.d.	-	2.73	0.16	5.05	0.58	3.43	0.22	0.76	0.01
		**Total**	**6.06**		**14.68**		**1.29**		**0.55**		**11.70**		**28.01**		**13.18**		**12.28**	
		***Aldehydes***																
508	2.31	Propanal	n.d.	-	n.d.	-	0.77	0.03	n.d.	-	0.96	0.04	1.10	0.08	0.97	0.05	n.d.	-
574	3.23	2-Methyl-2-propenal	n.d.	-	n.d.	-	0.61	0.03	n.d.	-	n.d.	-	n.d.	-	n.d.	-	n.d.	-
643	3.74	2-Methyl-butanal	1.73	0.02	0.56	0.02	n.d.	-	n.d.	-	n.d.	-	n.d.	-	n.d.	-	n.d.	-
643	3.83	3-Methyl-butanal	0.90	0.16	n.d.	-	n.d.	-	n.d.	-	n.d.	-	n.d.	-	n.d.	-	n.d.	-
785	10.12	Hexanal	0.70	0.02	5.57	0.14	10.15	0.29	n.d.	-	2.97	0.17	6.89	0.01	2.60	0.15	3.42	0.03
905	15.05	Heptanal	0.76	0.03	n.d.	-	1.03	0.03	n.d.	-	n.d.	-	n.d.	-	0.67	0.10	0.62	0.03
814	16.24	2-Hexenal	n.d.	-	n.d.	-	n.d.	-	n.d.	-	1.19	0.12	n.d.	-	1.41	0.03	n.d.	-
1005	18.74	Octanal	n.d.	-	n.d.	-	2.47	0.02	n.d.	-	2.03	0.23	17.40	0.16	1.87	0.03	n.d.	-
913	19.72	2-Heptenal	n.d.	-	3.10	0.05	2.61	0.22	n.d.	-	1.86	0.09	6.44	0.41	1.59	0.03	1.22	0.05
1104	21.67	Nonanal	0.73	0.16	n.d.	-	2.63	0.30	n.d.	-	n.d.	-	4.92	0.03	1.07	-	n.d.	-
1013	22.53	2-Octenal	n.d.	-	n.d.	-	0.68	0.05	n.d.	-	n.d.	-	2.07	0.08	n.d.	-	0.61	0.02
921	24.01	2,4-Heptadienal	n.d.	-	1.27	0.05	0.74	0.14	n.d.	-	n.d.	-	7.97	0.34	n.d.	-	n.d.	-
982	24.77	Benzaldehyde	n.d.	-	n.d.	-	n.d.	-	n.d.	-	1.86	0.01	25.84	0.45	n.d.	-	n.d.	-
1174	28.04	3,7-Dimethyl-2,6-octadienal	n.d.	-	n.d.	-	n.d.	-	n.d.	-	n.d.	-	44.49	4.40	n.d.	-	n.d.	-
		**Total**	**4.82**		**10.50**		**21.68**		**n.d.**		**10.87**		**117.14**		**10.19**		**5.88**	
		***Esters***																
487	2.63	Acetic acid-methyl ester	0.78	0.02	0.72	0.04	n.d.	-	n.d.	-	n.d.	-	n.d.	-	n.d.	-	n.d.	-
586	3.33	Acetic acid-ethyl ester	n.d.	-	8.70	0.12	302.74	12.32	n.d.	-	n.d.	-	n.d.	-	n.d.	-	n.d.	-
686	5.35	Acetic acid-propyl ester	n.d.	-	n.d.	-	1.45	0.07	n.d.	-	n.d.	-	n.d.	-	n.d.	-	n.d.	-
721	6.77	Acetic acid-2-methyl-propyl ester	n.d.	-	n.d.	-	5.39	0.06	n.d.	-	n.d.	-	n.d.	-	n.d.	-	n.d.	-
785	9.76	Acetic acid-buthyl ester	n.d.	-	n.d.	-	1.17	0.11	n.d.	-	n.d.	-	n.d.	-	n.d.	-	n.d.	-
820	12.27	1-Butanol-3-methyl acetate	n.d.	-	n.d.	-	7.46	0.17	n.d.	-	n.d.	-	16.98	0.21	n.d.	-	n.d.	-
992	19.62	3-Hexen-1-ol-acetate	n.d.	-	n.d.	-	n.d.	-	n.d.	-	n.d.	-	n.d.	-	0.64	0.02	n.d.	-
1183	22.24	Butanoic acid-hexyl ester	n.d.	-	n.d.	-	n.d.	-	0.60	0.02	n.d.	-	n.d.	-	n.d.	-	n.d.	-
		**Total**	**0.78**		**9.42**		**318.21**		**0.60**		**n.d.**		**16.98**		**0.64**		**n.d.**	
		***Ketones***																
455	2.50	2-Propanone	1.76	0.13	3.50	0.47	8.56	0.57	n.d.	-	2.46	0.51	5.03	0.65	1.78	0.14	0.96	0.01
1161	13.19	1-(1,3-dimethyl-3-cyclohexen-1-yl)-Ethanone	n.d.	-	1.89	0.18	n.d.	-	n.d.	-	n.d.	-	2.07	0.15	n.d.	-	0.90	0.06
853	14.89	2-Heptanone	n.d.	-	3.32	0.09	0.71	0.02	n.d.	-	2.69	0.16	n.d.	-	2.74	0.06	1.00	0.06
952	18.57	2-Octanone	n.d.	-	4.63	0.64	n.d.	-	n.d.	-	n.d.	-	n.d.	-	n.d.	-	n.d.	-
960	19.99	6-Octen-2-one	n.d.	-	0.86	0.11	n.d.	-	n.d.	-	n.d.	-	n.d.	-	n.d.	-	n.d.	-
987	20.17	6-Methyl-5 hepten-2 one	1.25	0.04	8.97	1.20	10.55	0.42	n.d.	-	2.28	0.11	17.04	0.14	1.63	0.00	2.26	0.13
960	21.96	3-Octen-2-one	n.d.	-	n.d.	-	n.d.	-	n.d.	-	n.d.	-	n.d.	-	n.d.	-	n.d.	-
962	22.51	Ketone	0.69	0.02	n.d.	-	n.d.	-	n.d.	-	n.d.	-	n.d.	-	n.d.	-	n.d.	-
968	24.67	3,5-Octadien-2-one	n.d.	-	n.d.	-	4.94	0.41	n.d.	-	n.d.	-	n.d.	-	n.d.	-	n.d.	-
		**Total**	**3.70**		**23.17**		**24.77**		**n.d.**		**7.44**		**24.14**		**6.15**		**5.12**	
		***Terpenes***																
939	6.67	α-Pinene	14.25	0.55	21.47	0.38	n.d.	-	73.23	9.63	112.95	1.47	5883.13	22.05	119.09	0.23	52.84	1.11
932	7.03	α-Thujene	0.98	0.17	1.18	0.08	n.d.	-	0.79	0.08	2.86	0.19	44.54	1.08	3.24	0.10	n.d.	-
961	8.67	Camphene	n.d.	-	n.d.	-	n.d.	-	0.94	0.27	1.57	0.09	65.97	3.91	1.60	0.01	0.95	0.03
989	10.75	β-Pinene	4.15	0.05	4.45	0.08	n.d.	-	23.36	4.71	35.65	2.06	625.28	4.35	37.08	0.48	17.91	0.28
985	11.66	Sabinene	n.d.	-	1.26	0.07	n.d.	-	n.d.	-	1.66	0.01	98.17	0.06	1.53	0.08	n.d.	-
879	11.74	2,4(10)-Thujadien	n.d.	-	0.85	0.00	n.d.	-	n.d.	-	n.d.	-	n.d.	-	n.d.	-	n.d.	-
1017	12.99	δ-3-Carene	1.73	0.03	0.61	0.09	n.d.	-	7.42	1.33	50.22	1.18	4.69	0.31	51.31	2.68	6.17	0.50
1015	13.81	α-Phellandrene	3.70	0.14	3.90	0.09	n.d.	-	2.91	0.65	6.78	0.01	4.97	0.04	8.06	0.18	1.85	0.09
991	14.24	β-Myrcene	108.13	0.09	34.63	1.00	9.14	0.48	419.53	33.32	389.37	20.60	2908.00	60.01	419.01	6.04	189.05	1.74
1026	14.44	α-Terpinene	3.60	0.38	10.01	0.56	n.d.	-	2.44	0.07	5.51	0.17	34.87	3.09	6.82	0.12	1.74	0.01
1038	15.31	Limonene	6.05	0.01	8.03	0.44	1.60	0.07	65.17	7.36	23.67	1.33	8841.42	171.34	20.17	0.26	23.97	0.34
1045	15.49	Eucalyptol	3.67	0.01	5.60	0.35	7.15	0.26	2.65	0.13	4.18	0.27	13.66	1.60	3.09	0.07	8.62	0.12
946	15.61	β-Phellandrene	7.16	0.14	4.80	0.52	n.d.	-	8.40	0.80	19.02	1.05	61.90	4.71	20.05	0.16	7.38	0.22
976	17.05	*Cis*-ocimene	0.48	0.02	2.17	0.17	0.48	0.00	16.55	1.07	21.17	0.88	7.13	0.24	19.24	0.23	5.50	0.11
1066	17.15	γ-Terpinene	5.38	0.01	5.84	0.58	0.59	0.06	2.11	0.09	4.80	0.23	499.58	9.26	4.61	0.08	2.48	0.04
1000	17.24	Terpene	n.d.	-	n.d.	-	2.48	0.24	n.d.	-	2.59	0.02	17.49	0.05	1.82	0.04	n.d.	-
1029	17.60	β-Ocimene	21.75	1.08	7.55	0.53	9.96	0.48	194.00	21.13	192.39	5.19	22.82	0.17	213.15	1.26	42.00	1.72
1034	18.01	*p*-Cymene	3.13	0.11	8.53	0.97	0.81	0.01	2.98	0.33	12.37	0.05	144.55	1.92	10.02	0.15	5.62	0.09
1094	18.43	α-Terpinolene	62.12	0.67	7.48	0.80	n.d.	-	111.31	14.58	265.95	16.97	33.73	0.11	297.20	3.11	73.16	3.13
1177	22.74	Para-cymenyl	13.97	0.88	11.28	0.16	n.d.	-	3.30	0.82	64.48	5.97	134.94	1.32	49.21	0.39	4.70	0.24
1136	23.01	Terpene	n.d.	-	n.d.	-	n.d.	-	1.55	0.36	1.39	0.11	n.d.	-	1.43	0.05	n.d.	-
1083	23.35	4,8-Epoxy-*p*-menth-1-ene	1.30	0.08	2.08	0.23	n.d.	-	2.22	0.03	6.44	0.16	3.08	0.03	5.20	0.60	1.32	0.01
1164	23.48	Linalool oxide	n.d.	-	n.d.	-	n.d.	-	n.d.	-	1.11	0.03	17.95	0.94	1.51	0.01	n.d.	-
1221	23.68	α-Ylangene	n.d.	-	1.09	0.23	n.d.	-	n.d.	-	0.52	0.00	2.19	0.09	n.d.	-	1.18	0.07
1082	25.29	β-Linalool	5.52	0.81	2.22	0.32	10.55	1.27	0.82	0.01	5.19	0.01	1471.75	15.90	5.36	0.37	n.d.	-
1494	25.75	γ-Caryophyllene	n.d.	-	5.87	1.68	2.02	0.24	1.03	0.06	1.81	0.05	30.72	1.10	1.32	0.04	4.40	0.17
1430	26.03	α-Bergamotene	2.42	0.21	11.89	3.95	1.75	0.08	2.21	0.19	2.31	0.02	40.96	0.21	1.01	0.08	9.44	0.07
1456	26.12	α-Guaiene	2.46	0.05	n.d.	-	7.19	0.57	n.d.	-	n.d.	-	5.20	0.18	n.d.	-	n.d.	-
1494	26.23	*Trans*-caryophyllene	17.34	1.81	159.92	49.08	90.68	7.15	40.67	3.44	48.77	1.93	425.63	7.84	32.06	4.48	110.41	1.33
		***Terpenes***																
1209	26.40	4-Terpineol	2.32	0.23	3.34	0.62	0.55	0.06	n.d.	-	2.80	0.24	15.34	1.66	1.71	0.18	n.d.	-
1440	26.59	Sesquiterpene	n.d.	-	3.03	0.70	1.37	0.18	n.d.	-	1.64	0.07	17.73	0.11	n.d.	-	1.13	0.10
1386	27.21	Sesquiterpene	n.d.	-	6.09	2.17	n.d.	-	n.d.	-	0.58	0.05	12.62	0.59	n.d.	-	7.01	0.26
1131	27.47	*Trans*-pinocarveol	n.d.	-	9.69	1.69	n.d.	-	n.d.	-	3.04	0.07	10.99	0.49	2.30	0.08	5.12	0.08
1482	27.73	α-Humulene	6.28	0.44	45.95	15.88	21.61	1.49	10.13	1.54	9.62	0.69	117.14	3.27	5.94	0.88	32.25	1.15
1189	28.13	1,8-Menthadien-4-ol	6.23	1.02	21.18	4.16	n.d.	-	2.19	0.43	19.68	0.44	44.91	2.39	12.61	0.58	6.86	0.41
1209	28.32	α-Terpineol	2.79	0.43	3.31	0.85	1.13	0.02	n.d.	-	2.90	0.24	14.35	1.10	1.83	0.04	0.63	0.07
1189	28.40	Borneol	0.52	0.04	2.78	0.74	n.d.	-	n.d.	-	0.64	0.04	1.59	2.25	n.d.	-	n.d.	-
1490	28.65	δ-Guaiene	1.56	0.01	n.d.	-	n.d.	-	n.d.	-	n.d.	-	n.d.	-	n.d.	-	n.d.	-
1519	28.72	β-Selinene	0.85	0.01	11.64	4.28	1.65	0.02	0.96	0.19	1.46	0.13	41.02	1.77	0.85	0.17	8.15	0.36
1522	28.82	α-Selinene	1.04	0.01	7.51	2.69	1.06	0.14	n.d.	-	1.04	0.06	26.41	1.64	n.d.	-	4.51	0.20
1474	28.98	Sesquiterpene	n.d.	-	1.88	0.58	n.d.	-	n.d.	-	n.d.	-	53.97	6.99	n.d.	-	0.92	0.02
1190	29.03	Carvone	n.d.	-	n.d.	-	n.d.	-	n.d.	-	n.d.	-	n.d.	-	n.d.	-	n.d.	-
1507	29.86	Selina-3,7(11)-diene	n.d.	-	6.79	2.41	n.d.	-	0.96	0.31	1.85	0.06	25.33	2.61	n.d.	-	3.78	0.21
1191	30.13	Myrtenol	n.d.	-	1.49	0.26	n.d.	-	n.d.	-	0.94	0.04	3.18	0.40	n.d.	-	0.80	0.03
1284	31.17	Cuminol	4.00	0.64	7.44	1.98	n.d.	-	0.54	0.07	7.87	0.61	30.26	3.84	7.02	0.08	1.46	0.10
1322	33.41	Humulene oxide	n.d.	-	2.22	0.92	0.72	0.13	n.d.	-	n.d.	-	n.d.	-	n.d.	-	1.36	0.14
1419	34.11	Sesquiterpene	n.d.	-	n.d.	-	1.54	0.03	n.d.	-	n.d.	-	n.d.	-	n.d.	-	n.d.	-
1392	34.86	Eugenol	n.d.	-	n.d.	-	n.d.	-	n.d.	-	0.82	0.00	1.12	0.05	1.17	0.02	n.d.	-
		**Total**	**314.90**		**457.03**		**174.05**		**1000.37**		**1339.56**		**21860.3**		**1367.63**		**644.70**	
		***Miscellaneous***																
906	11.03	3,3,6-Trimethyl-1,5-heptadiene	n.d.	-	n.d.	-	n.d.	-	n.d.	-	n.d.	-	11.87	0.33	n.d.	-	n.d.	-
907	12.72	1,3-Dimethyl-benzene	n.d.	-	0.96	0.08	n.d.	-	2.17	0.38	n.d.	-	1.41	0.02	n.d.	-	n.d.	-
1040	16.87	2-Pentyl-furan	n.d.	-	1.82	0.20	n.d.	-	n.d.	-	0.24	0.01	3.94	0.10	n.d.	-	1.99	0.07
1020	18.31	1,2,4,-Trimethyl-benzene	n.d.	-	n.d.	-	1.35	0.38	n.d.	-	n.d.	-	n.d.	-	n.d.	-	n.d.	-
894	19.54	2,5-Dimethyl-pyrazine	1.03	0.05	n.d.	-	n.d.	-	n.d.	-	n.d.	-	1.23	0.04	n.d.	-	n.d.	-
891	19.74	2,6-Dimethyl-pyrazine	0.82	0.03	n.d.	-	n.d.	-	n.d.	-	n.d.	-	n.d.	-	n.d.	-	n.d.	-
1176	20.72	1,4-Bis (1-methylethyl)-benzene	n.d.	-	n.d.	-	n.d.	-	n.d.	-	n.d.	-	1.81	0.01	n.d.	-	n.d.	-
985	21.89	2,6-Dimethyl-2,6-octadiene	n.d.	-	n.d.	-	3.25	0.14	n.d.	-	n.d.	-	n.d.	-	n.d.	-	n.d.	-
1081	22.10	Diethyl carbitol	n.d.	-	n.d.	-	n.d.	-	n.d.	-	n.d.	-	n.d.	-	n.d.	-	n.d.	-
1039	22.36	1,3,5-Trimethylenecycloheptane	n.d.	-	1.07	0.26	n.d.	-	0.80	0.12	1.52	0.00	2.62	0.06	1.70	0.02	n.d.	-
986	28.45	5-Ethyldihydro-2(3H)-furanone	n.d.	-	1.92	0.14	n.d.	-	n.d.	-	3.89	0.41	27.39	2.75	6.63	0.41	0.96	0.15
1190	30.85	1-Methoxy-4(1-propenyl)-benzene	n.d.	-	n.d.	-	n.d.	-	3.22	0.07	n.d.	-	n.d.	-	n.d.	-	n.d.	-
		**Total**	**1.85**		**7.19**		**4.60**		**6.19**		**8.38**		**54.23**		**10.93**		**2.95**	
		**Oil Samples**	**9**		**10**		**11**		**12**		**13**		**14**		**15**			
		**Matrix**	**Hemp Seed Oil**		**Olive Oil**		**Hemp Seed Oil**		**Hemp Seed Oil**		**Olive Oil**		**Hemp Seed Oil**		**Hemp Seed Oil**			
**RI ^a^**	**R.T ^b^**	**Compound**	**Average ^c^** **μg/g**	**SD** **(±)**	**Average ^c^** **μg/g**	**SD** **(±)**	**Average ^c^** **μg/g**	**SD** **(±)**	**Average ^c^** **μg/g**	**SD** **(±)**	**Average ^c^** **μg/g**	**SD** **(±)**	**Average ^c^** **μg/g**	**SD** **(±)**	**Average^c^** **μg/g**	**SD** **(±)**		
		***Alcohols***																
831	20.63	1-Hexanol	6.19	0.32	2.66	0.08	2.00	0.14	4.37	0.20	0.67	0.02	3.52	0.23	9.67	0.16		
868	21.43	3-Hexen-1-ol	n.d.	-	1.81	0.03	n.d.	-	n.d.	-	0.65	0.01	0.86	0.06	n.d.	-		
849	22.02	2-Hexen-1-ol	n.d.	-	1.15	0.05	n.d.	-	0.62	0.07	0.63	0.00	n.d.	-	n.d.	-		
969	23.07	1-Octen-3-ol	1.08	0.03	0.79	0.01	1.26	0.13	1.23	0.07	n.d.	-	1.35	0.00	n.d.	-		
960	23.17	1-Heptanol	n.d.	-	n.d.	-	n.d.	-	0.72	0.04	n.d.	-	n.d.	-	n.d.	-		
1059	25.48	1-Octanol	n.d.	-	0.58	0.11	n.d.	-	n.d.	-	n.d.	-	2.30	0.25	n.d.	-		
1068	27.98	3,3,6-Trimethyl-1,5-heptadien-4-ol	n.d.	-	n.d.	-	n.d.	-	n.d.	-	n.d.	-	1.56	0.10	n.d.	-		
1036	31.59	α-Toluenol	n.d.	-	3.03	0.35	n.d.	-	1.39	0.15	n.d.	-	2.08	0.61	n.d.	-		
1136	32.06	Benzeneethanol	0.62	0.03	3.07	0.62	n.d.	-	1.25	0.01	0.87	0.13	2.72	0.98	n.d.	-		
		**Total**	**7.89**		**13.09**		**3.26**		**9.57**		**2.82**		**14.38**		**9.67**			
		***Aldehydes***																
508	2.31	Propanal	n.d.	-	1.04	0.00	n.d.	-	n.d.	-	0.97	0.02	0.62	0.10	n.d.	-		
574	3.23	2-Methyl-2-propenal	n.d.	-	n.d.	-	n.d.	-	n.d.	-	n.d.	-	n.d.	-	n.d.	-		
643	3.74	2-Methyl-butanal	n.d.	-	n.d.	-	n.d.	-	n.d.	-	n.d.	-	n.d.	-	n.d.	-		
643	3.83	3-Methyl-butanal	n.d.	-	n.d.	-	n.d.	-	n.d.	-	n.d.	-	n.d.	-	n.d.	-		
785	10.12	Hexanal	6.35	0.40	2.53	0.01	10.51	0.36	7.04	0.29	3.18	0.36	2.60	0.18	1.80	0.12		
905	15.05	Heptanal	n.d.	-	0.63	0.03	0.46	0.02	n.d.	-	n.d.	-	n.d.	-	n.d.	-		
814	16.24	2-Hexenal	1.23	0.06	1.41	0.03	1.02	0.02	0.53	0.75	11.50	0.40	0.82	0.04	n.d.	-		
1005	18.74	Octanal	n.d.	-	1.92	0.07	n.d.	-	1.93	0.32	n.d.	-	n.d.	-	n.d.	-		
913	19.72	2-Heptenal	1.17	0.05	1.72	0.09	5.21	0.57	8.29	0.77	1.54	0.00	5.44	0.12	1.11	0.02		
1104	21.67	Nonanal	n.d.	-	1.20	0.14	n.d.	-	n.d.	-	n.d.	-	n.d.	-	n.d.	-		
1013	22.53	2-Octenal	1.49	0.02	n.d.	-	n.d.	-	0.95	0.00	n.d.	-	n.d.	-	n.d.	-		
921	24.01	2,4-Heptadienal	8.05	0.11	n.d.	-	2.31	0.19	1.37	0.20	n.d.	-	1.24	0.02	n.d.	-		
982	24.77	Benzaldehyde	1.45	0.18	n.d.	-	1.90	1.67	1.55	0.31	0.43	0.10	8.46	3.09	n.d.	-		
1174	28.04	3,7-Dimethyl-2,6-octadienal	n.d.	-	n.d.	-	n.d.	-	n.d.	-	n.d.	-	1.30	0.07	n.d.	-		
		**Total**	**19.73**		**10.43**		**21.41**		**21.66**		**17.61**		**20.48**		**2.92**			
487	2.63	Acetic acid-methyl ester	n.d.	-	n.d.	-	n.d.	-	n.d.	-	n.d.	-	n.d.	-	n.d.	-		
586	3.33	Acetic acid-ethyl ester	n.d.	-	n.d.	-	n.d.	-	n.d.	-	n.d.	-	n.d.	-	n.d.	-		
686	5.35	Acetic acid-propyl ester	n.d.	-	n.d.	-	n.d.	-	n.d.	-	n.d.	-	n.d.	-	n.d.	-		
721	6.77	Acetic acid-2-methyl-propyl ester	n.d.	-	n.d.	-	n.d.	-	n.d.	-	n.d.	-	n.d.	-	n.d.	-		
785	9.76	Acetic acid-buthyl ester	n.d.	-	n.d.	-	n.d.	-	n.d.	-	n.d.	-	n.d.	-	n.d.	-		
820	12.27	1-Butanol-3-methyl acetate	n.d.	-	n.d.	-	n.d.	-	n.d.	-	n.d.	-	n.d.	-	n.d.	-		
992	19.62	3-Hexen-1-ol-acetate	n.d.	-	0.66	0.01	n.d.	-	n.d.	-	0.82	0.08	n.d.	-	n.d.	-		
1183	22.24	Butanoic acid-hexyl ester	n.d.	-	0.53	0.04	n.d.	-	n.d.	-	n.d.	-	n.d.	-	n.d.	-		
		**Total**	**n.d.**		**1.19**		**n.d.**		**n.d.**		**0.82**		**n.d.**		**n.d.**			
		***Ketones***																
455	2.50	2-Propanone	0.63	0.04	2.20	0.24	0.54	0.05	2.11	0.02	0.78	0.15	2.41	0.45	n.d.	-		
1161	13.19	1-(1,3-dimethyl-3-cyclohexen-1-yl)-Ethanone	n.d.	-	n.d.	-	n.d.	-	2.75	0.05	n.d.	-	0.89	0.05	n.d.	-		
853	14.89	2-Heptanone	n.d.	-	2.70	0.03	1.05	0.04	1.32	0.02	n.d.	-	1.45	0.36	n.d.	-		
952	18.57	2-Octanone	n.d.	-	n.d.	-	5.08	0.42	0.55	0.03	n.d.	-	n.d.	-	n.d.	-		
960	19.99	6-Octen-2-one	n.d.	-	n.d.	-	0.80	0.04	n.d.	-	n.d.	-	n.d.	-	n.d.	-		
987	20.17	6-Methyl-5 hepten-2 one	0.73	0.10	1.65	0.06	0.53	0.03	6.00	0.58	0.76	0.04	6.50	0.28	n.d.	-		
960	21.96	3-Octen-2-one	n.d.	-	n.d.	-	0.57	0.05	n.d.	-	n.d.	-	n.d.	-	n.d.	-		
962	22.51	Ketone	n.d.	-	n.d.	-	n.d.	-	n.d.	-	n.d.	-	0.71	0.01	n.d.	-		
968	24.67	3,5-Octadien-2-one	0.59	0.07	n.d.	-	1.44	0.17	1.44	0.13	n.d.	-	1.39	0.20	n.d.	-		
		**Total**	**1.94**		**6.55**		**10.01**		**14.16**		**1.54**		**13.35**		**n.d.**			
		***Terpenes***																
939	6.67	α-Pinene	1.35	0.06	120.21	0.54	4.27	0.25	147.07	1.23	n.d.	-	94.47	3.96	2.27	0.07		
932	7.03	α-Thujene	n.d.	-	2.86	0.13	n.d.	-	9.40	0.18	n.d.	-	2.16	0.11	n.d.	-		
961	8.67	Camphene	n.d.	-	1.69	0.15	n.d.	-	3.04	0.02	n.d.	-	7.45	0.26	n.d.	-		
989	10.75	β-Pinene	0.70	0.00	37.67	1.33	1.30	0.14	33.28	0.36	n.d.	-	21.91	0.34	0.87	0.01		
985	11.66	Sabinene	n.d.	-	1.64	0.13	n.d.	-	0.71	0.05	n.d.	-	n.d.	-	n.d.	-		
879	11.74	2,4(10)-Thujadien	n.d.	-	n.d.	-	n.d.	-	n.d.	-	n.d.	-	n.d.	-	n.d.	-		
1017	12.99	δ-3-Carene	n.d.	-	48.70	4.87	n.d.	-	3.82	0.10	n.d.	-	6.63	0.99	n.d.	-		
1015	13.81	α-Phellandrene	n.d.	-	7.37	0.11	n.d.	-	2.59	0.40	n.d.	-	3.70	0.60	n.d.	-		
991	14.24	β-Myrcene	13.88	2.66	429.64	5.70	10.12	0.33	344.78	5.22	n.d.	-	74.69	5.07	10.65	0.30		
1026	14.44	α-Terpinene	n.d.	-	4.29	0.26	n.d.	-	2.35	0.19	n.d.	-	2.95	0.49	n.d.	-		
1038	15.31	Limonene	2.38	0.50	20.56	0.29	2.40	0.15	50.09	1.95	n.d.	-	23.56	1.63	5.53	0.46		
1045	15.49	Eucalyptol	n.d.	-	3.13	0.02	0.41	0.06	15.41	0.72	0.56	0.09	17.91	0.72	n.d.	-		
946	15.61	β-Phellandrene	n.d.	-	20.96	0.14	n.d.	-	7.16	0.27	n.d.	-	13.75	0.51	n.d.	-		
976	17.05	*Cis*-ocimene	n.d.	-	19.76	0.13	n.d.	-	5.45	0.45	n.d.	-	7.22	0.43	n.d.	-		
1066	17.15	γ-Terpinene	n.d.	-	4.77	0.03	n.d.	-	7.17	0.43	n.d.	-	4.59	0.24	n.d.	-		
		***Terpenes***																
1000	17.24	Terpene	0.87	0.01	1.88	0.14	n.d.	-	n.d.	-	n.d.	-	1.30	0.17	n.d.	-		
1029	17.60	β-Ocimene	4.44	0.07	217.33	2.36	2.42	0.28	40.93	2.46	0.88	0.01	24.18	1.39	2.91	0.27		
1034	18.01	p-Cymene	n.d.	-	10.17	0.39	0.66	0.05	43.02	2.52	0.66	0.27	13.51	0.89	n.d.	-		
1094	18.43	α-Terpinolene	3.55	0.19	249.23	0.57	0.76	0.07	52.02	3.15	n.d.	-	16.01	1.06	1.00	0.07		
1177	22.74	Para-cymenyl	1.81	0.11	47.64	0.55	0.56	0.20	7.84	0.51	1.07	0.02	7.16	0.93	n.d.	-		
1136	23.01	Terpene	n.d.	-	1.67	0.11	n.d.	-	0.53	0.06	n.d.	-	n.d.	-	n.d.	-		
1083	23.35	4,8-Epoxy-*p*-menth-1-ene	n.d.	-	5.65	0.01	n.d.	-	2.49	0.35	n.d.	-	n.d.	-	n.d.	-		
1164	23.48	Linalool oxide	0.70	0.01	1.37	0.40	n.d.	-	2.96	0.25	n.d.	-	n.d.	-	n.d.	-		
1221	23.68	α-Ylangene	n.d.	-	n.d.	-	n.d.	-	n.d.	-	n.d.	-	n.d.	-	n.d.	-		
1082	25.29	β-Linalool	n.d.	-	6.35	0.07	n.d.	-	3.98	0.45	n.d.	-	4.90	0.14	n.d.	-		
1494	25.75	γ-Caryophyllene	8.60	1.21	1.63	0.09	0.57	0.00	5.30	0.59	1.23	0.05	8.86	1.07	n.d.	-		
1430	26.03	α-Bergamotene	6.78	1.09	1.06	0.24	0.53	0.03	11.82	1.57	2.94	0.11	19.89	3.59	n.d.	-		
1456	26.12	α-Guaiene	n.d.	-	n.d.	-	n.d.	-	n.d.	-	n.d.	-	1.08	0.19	n.d.	-		
1494	26.23	*Trans*-caryophyllene	78.99	10.43	39.18	2.69	4.72	0.14	92.75	9.99	15.78	0.51	228.18	33.12	1.62	0.28		
1209	26.40	4-Terpineol	n.d.	-	2.07	0.13	n.d.	-	3.74	0.68	n.d.	-	2.46	0.32	n.d.	-		
1440	26.59	Sesquiterpene	n.d.	-	n.d.	-	n.d.	-	2.50	0.02	n.d.	-	2.80	1.76	n.d.	-		
1386	27.21	Sesquiterpene	1.73	0.24	n.d.	-	n.d.	-	6.03	0.75	1.56	0.03	7.09	1.10	n.d.	-		
1131	27.47	Trans-pinocarveol	n.d.	-	2.74	0.03	n.d.	-	8.40	0.80	0.85	0.10	2.41	0.17	n.d.	-		
1482	27.73	α-Humulene	31.19	5.31	7.35	0.58	1.28	0.04	27.08	3.69	5.27	0.05	68.14	12.59	n.d.	-		
1189	28.13	1,8-Menthadien-4-ol	2.13	0.13	15.23	1.04	1.44	0.81	12.22	1.05	2.44	0.08	19.06	0.12	n.d.	-		
1209	28.32	α-Terpineol	n.d.	-	2.13	0.19	n.d.	-	3.26	0.03	0.76	0.12	3.59	1.20	n.d.	-		
1189	28.40	Borneol	n.d.	-	n.d.	-	n.d.	-	1.13	0.09	n.d.	-	n.d.	-	n.d.	-		
1490	28.65	δ-Guaiene	n.d.	-	n.d.	-	n.d.	-	n.d.	-	n.d.	-	n.d.	-	n.d.	-		
1519	28.72	β-Selinene	6.81	1.46	0.90	0.15	n.d.	-	8.70	1.17	3.03	0.03	13.72	2.84	n.d.	-		
1522	28.82	α-Selinene	3.53	0.59	1.05	0.12	n.d.	-	5.44	0.74	1.81	0.05	9.48	1.82	n.d.	-		
1474	28.98	Sesquiterpene	1.60	0.24	n.d.	-	n.d.	-	1.01	0.15	0.58	0.04	2.13	0.41	n.d.	-		
1190	29.03	Carvone	n.d.	-	n.d.	-	n.d.	-	n.d.	-	n.d.	-	n.d.	-	0.79	0.14		
1507	29.86	Selina-3,7(11)-diene	5.95	1.25	1.88	0.33	n.d.	-	n.d.	-	1.86	0.03	6.99	1.18	n.d.	-		
1191	30.13	Myrtenol	n.d.	-	n.d.	-	n.d.	-	1.23	0.16	n.d.	-	n.d.	-	n.d.	-		
1284	31.17	Cuminol	1.51	0.26	8.56	1.41	n.d.	-	3.13	0.07	0.97	0.11	6.46	1.79	n.d.	-		
1322	33.41	Humulene oxide	1.71	0.47	n.d.	-	n.d.	-	1.54	0.32	0.92	0.05	2.44	0.37	n.d.	-		
1419	34.11	Sesquiterpene	n.d.	-	n.d.	-	n.d.	-	n.d.	-	n.d.	-	n.d.	-	n.d.	-		
1392	34.86	Eugenol	0.76	0.15	1.18	0.14	n.d.	-	n.d.	-	n.d.	-	n.d.	-	n.d.	-		
		**Total**	**180.97**		**1349.52**		**31.43**		**981.37**		**43.15**		**752.82**		**25.64**			
		***Miscellaneous***																
906	11.03	3,3,6-Trimethyl-1,5-heptadiene	n.d.	-	n.d.	-	n.d.	-	n.d.	-	n.d.	-	n.d.	-	n.d.	-		
907	12.72	1,3-Dimethyl-benzene	n.d.	-	n.d.	-	n.d.	-	n.d.	-	n.d.	-	n.d.	-	n.d.	-		
1040	16.87	2-Pentyl-furan	3.01	0.27	n.d.	-	0.56	0.04	n.d.	-	n.d.	-	1.26	0.12	n.d.	-		
1020	18.31	1,2,4,-Trimethyl-benzene	n.d.	-	n.d.	-	n.d.	-	n.d.	-	n.d.	-	n.d.	-	n.d.	-		
894	19.54	2,5-Dimethyl-pyrazine	n.d.	-	n.d.	-	n.d.	-	n.d.	-	n.d.	-	n.d.	-	n.d.	-		
891	19.74	2,6-Dimethyl-pyrazine	n.d.	-	n.d.	-	n.d.	-	n.d.	-	n.d.	-	n.d.	-	n.d.	-		
1176	20.72	1,4-Bis (1-methylethyl)-benzene	n.d.	-	n.d.	-	n.d.	-	n.d.	-	n.d.	-	n.d.	-	n.d.	-		
985	21.89	2,6-Dimethyl-2,6-octadiene	n.d.	-	n.d.	-	n.d.	-	n.d.	-	n.d.	-	n.d.	-	n.d.	-		
1081	22.10	Diethyl carbitol	n.d.	-	n.d.	-	n.d.	-	n.d.	-	n.d.	-	n.d.	-	0.91	0.23		
1039	22.36	1,3,5-Trimethylenecycloheptane	n.d.	-	1.54	0.00	n.d.	-	n.d.	-	n.d.	-	1.15	0.06	n.d.	-		
986	28.45	5-Ethyldihydro-2(3H)-furanone	0.82	0.04	5.86	1.44	0.73	0.19	1.26	0.10	n.d.	-	5.40	1.36	n.d.	-		
1190	30.85	1-Methoxy-4(1-propenyl)-benzene	n.d.	-	n.d.	-	n.d.	-	n.d.	-	n.d.	-	n.d.	-	n.d.	-		

RI ^a^: retention index calculated on a Rtx-Wax (30 m × 0.25 mm × 0.25 μm f.t.); RT ^b^: retention time (min); Average ^c^: mean value (*n* = 3); Data are expressed in μg/g SD ^d^: Standard deviatio ; n.d.: not detected.

**Table 3:** Declared CBD content, oil matrix used and origin of the analysed CBD oil samples.

Samples	CBD Declared Content (%, *w*/*w*)	Matrix	Origin	Extraction Method (When Indicated)
Oil_1	/	Olive oil	Italy	Calvi et al., 2018
Oil_2	4	Hemp seed oil	Switzerland	ND *
Oil_3	1	Olive oil	Switzerland	ND
Oil_4	5	Caprylic/Capric Triglyceride (MCT)	Italy	ND
Oil_5	4	Olive oil	Switzerland	CO_2_ supercritical
Oil_6	3	Hemp oil	The Netherlands	CO_2_ supercritical
Oil_7	4	Olive oil	Spain	ND
Oil_8	3	Hemp seed oil	The Netherlands	ND
Oil_9	3	Hemp seed oil	The Netherlands	ND
Oil_10	4	Olive oil	The Netherlands	ND
Oil_11	/	Hemp seed oil	Switzerland	ND
Oil_12	2	Hemp seed oil	Switzerland	CO_2_ supercritical
Oil_13	4	Olive oil	France	ND
Oil_14	5	Hemp seed oil	Slovenia	CO_2_ supercritical
Oil_15	3	Hemp seed oil	UK	ND

* ND—not declared.
